# Alteration of Proteomes in First-Generation Cultures of Bacillus pumilus Spores Exposed to Outer Space

**DOI:** 10.1128/mSystems.00195-19

**Published:** 2019-06-11

**Authors:** Abby J. Chiang, Ganesh Babu Malli Mohan, Nitin K. Singh, Parag A. Vaishampayan, Markus Kalkum, Kasthuri Venkateswaran

**Affiliations:** a Department of Molecular Imaging and Therapy, Beckman Research Institute of City of Hope, Duarte, California, USA; b Irell and Manella Graduate School of Biological Sciences, Beckman Research Institute of City of Hope, Duarte, California, USA; c Biotechnology and Planetary Protection Group, Jet Propulsion Laboratory, California Institute of Technology, Pasadena, California, USA; Princeton University

**Keywords:** *Bacillus pumilus* SAFR-032, Mars atmosphere, proteomics, space conditions, tandem mass tag, UV resistance

## Abstract

Bacillus pumilus SAFR-032 was originally isolated from the Jet Propulsion Lab Spacecraft Assembly Facility and thoroughly characterized for its enhanced resistance to UV irradiation and oxidative stress. This unusual resistance of SAFR-032 is of particular concern in the context of planetary protection and calls for development of novel disinfection techniques to prevent extraterrestrial contamination. Previously, spores of SAFR-032 were exposed for 18 months to a variety of space conditions on board the International Space Station to investigate their resistance to Mars-like conditions and space travel. Here, proteomic characterization of vegetative SAFR-032 cells from space-surviving spores is presented in comparison to a ground control. Vegetative cells of the first passage were processed and subjected to quantitative proteomics using tandem mass tags. Approximately 60% of all proteins encoded by SAFR-032 were identified, and 301 proteins were differentially expressed among the strains. We found that proteins predicted to be involved in carbohydrate transport/metabolism and energy production/conversion had lower abundance than those of the ground control. For three proteins, we showed that the expected metabolic activities were decreased, as expected with direct enzymatic assays. This was consistent with a decrease of ATP production in the space-surviving strains. The same space-surviving strains showed increased abundance of proteins related to survival, growth advantage, and stress response. Such alterations in the proteomes provide insights into possible molecular mechanisms of *B. pumilus* SAFR-032 to adapt to and resist extreme extraterrestrial environments.

**IMPORTANCE** Spore-forming bacteria are well known for their resistance to harsh environments and are of concern for spreading contamination to extraterrestrial bodies during future life detection missions. Bacillus pumilus has been regularly isolated from spacecraft-associated surfaces and exhibited unusual resistance to ultraviolet light and other sterilization techniques. A better understanding of the mechanisms of microbial survival and enhanced resistance is essential for developing novel disinfection protocols for the purpose of planetary protection. While genomic analyses did not reveal the unique characteristics that explain elevated UV resistance of space-exposed *B. pumilus*, the proteomics study presented here provided intriguing insight on key metabolic changes. The observed proteomics aberrations reveal a complex biological phenomenon that plays a role in bacterial survival and adaptation under long-term exposure to outer space. This adaptive ability of microorganisms needs to be considered by those tasked with eliminating forward contamination.

## Abstract (precis)

Spore-forming bacteria are well known for their resistance to harsh environments and are of concern for spreading contamination to extraterrestrial bodies during future life detection missions. Bacillus pumilus has been regularly isolated from spacecraft-associated surfaces and exhibited unusual resistance to ultraviolet light and other sterilization techniques. A better understanding of the mechanisms of microbial survival and enhanced resistance is essential for developing novel disinfection protocols for the purpose of planetary protection. While genomic analyses did not reveal the unique characteristics that explain elevated UV resistance of space-exposed *B. pumilus*, the proteomics study presented here provided intriguing insight on key metabolic changes. The observed proteomics aberrations reveal a complex biological phenomenon that plays a role in bacterial survival and adaptation under long-term exposure to outer space. This adaptive ability of microorganisms needs to be considered by those tasked with eliminating forward contamination.

## INTRODUCTION

The hardy spores of *Bacillus* species are well known for their resistance to unfavorable conditions such as UV/gamma radiation, heat, desiccation, chemical disinfection, and starvation (1). In particular, the Bacillus pumilus SAFR-032 strain, which was originally isolated from the Jet Propulsion Lab Spacecraft Assembly Facility (JPL-SAF), exhibits unusually high resistance to UV radiation and peroxide treatment compared to other *Bacillus* species (2, 3). By comparing *B. pumilus* SAFR-032 with other *Bacillus* species isolated from SAF or obtained from various sources, spores of *B. pumilus* SAFR-032 exhibited at least 6-fold more resistance to UV irradiation than others (4). Genomic studies have been carried out to identify candidate genes that may be responsible for the enhanced stress resistance of *B. pumilus* SAFR-032. Compared to B. subtilis, several genes involved in spore coat formation and regulation, DNA repair, and peroxide resistance are absent in *B. pumilus* SAFR-032 (2, 5). However, several unique gene candidates involved in DNA repair and H_2_O_2_ neutralization were identified in SAFR-032 only. Nevertheless, the mechanisms that contribute to the survival and elevated resistance of SAFR-032 spores still need to be investigated in more detail to help find a new way to eliminate these spore-forming bacteria associated with spacecraft surfaces to prevent extraterrestrial contamination.

One goal of the PROTECT spaceflight experiment during the EXPOSE-E mission was to investigate the molecular mechanisms of resistance of wild-type *Bacillus* endospores to relevant outer space environments (6). The PROTECT experiment exposed spores of *B. pumilus* SAFR-032 to different extraterrestrial conditions on board the International Space Station (ISS) for 18 months. The space-exposed conditions included selected conditions of outer space (Space), simulated Martian surface conditions (Mars), and conditions both with and without solar exposure (UV and Dark, respectively). The detailed test parameters of each group (UV-Space, UV-Mars, Dark-Space, and Dark-Mars) were reported previously (6, 7). In summary, the exposure included space vacuum (∼10^−4 ^Pa), simulated Mars atmosphere (∼10^3^ Pa, 1.6% argon, 0.15% oxygen, 2.7% nitrogen in CO_2_), galactic cosmic radiation (140 to 155 mGy), temperature fluctuation (−20°C to +59.6°C), and either the full spectrum of solar extraterrestrial electromagnetic radiation (λ > 110 nm, 550 MJ/m^2^) or a simulated Mars UV radiation (λ > 200 nm, 400 MJ/m^2^) (6, 7). After 18 months of exposure in the EXPOSE facility, SAFR-032 spores from Dark-Space had a 10 to 40% survival rate, and the spores from Dark-Mars had 85 to 100% survival. In contrast, UV exposure resulted in nearly complete destruction of all spores, and only 19 colonies were isolated from test materials that survived the UV-Space and UV-Mars conditions (6). These space-surviving SAFR-032 spores were regrown after returning to Earth and were archived in 50 vials. We refer to this set of isolates as the parent isolates. First-generation cultures of vegetative cells from the parent space-surviving spores were studied. In comparison to the SAFR-032 ground control, the spores retrieved from the UV-Space and UV-Mars conditions showed the highest levels of UVC resistance. Interestingly, the vegetative cells of all space-surviving strains also showed a higher resistance of UVC compared to the ground control, but the vegetative cells of the Dark-Space-exposed conditions had the highest level of resistance among all of the space-surviving strains (6).

The objective of the present study was to gain insights into the mechanism of resistance and survival of outer space-exposed *B. pumilus* SAFR-032 using quantitative proteomics. Vegetative cells that were cultured from the first-generation colonies of space-surviving strains showed a higher radiation resistance than the ground control. It is therefore important to determine the differences in protein abundance under normal, unstressed growth conditions to better understand how the bacteria changed following 18 months of space exposure. Thus, quantitative protein abundances of these first-generation space-surviving strains were compared to the protein abundances in the SAFR-032 ground control. Multiplexed isobaric tandem mass tag (TMT) labeling allowed identification and quantification of the relative abundance of proteins across all strains. In this communication, we present quantitative changes in the bacterial proteome that are related not only to the essential catabolic pathways but also to those involved in bacterial survival, growth advantage, and stress response. These changes may contribute to the unique resistance characteristics of space-surviving strains.

## RESULTS AND DISCUSSION

### UV resistance of the space-exposed *B. pumilus* SAFR-032 strains.

**FIG 1:**
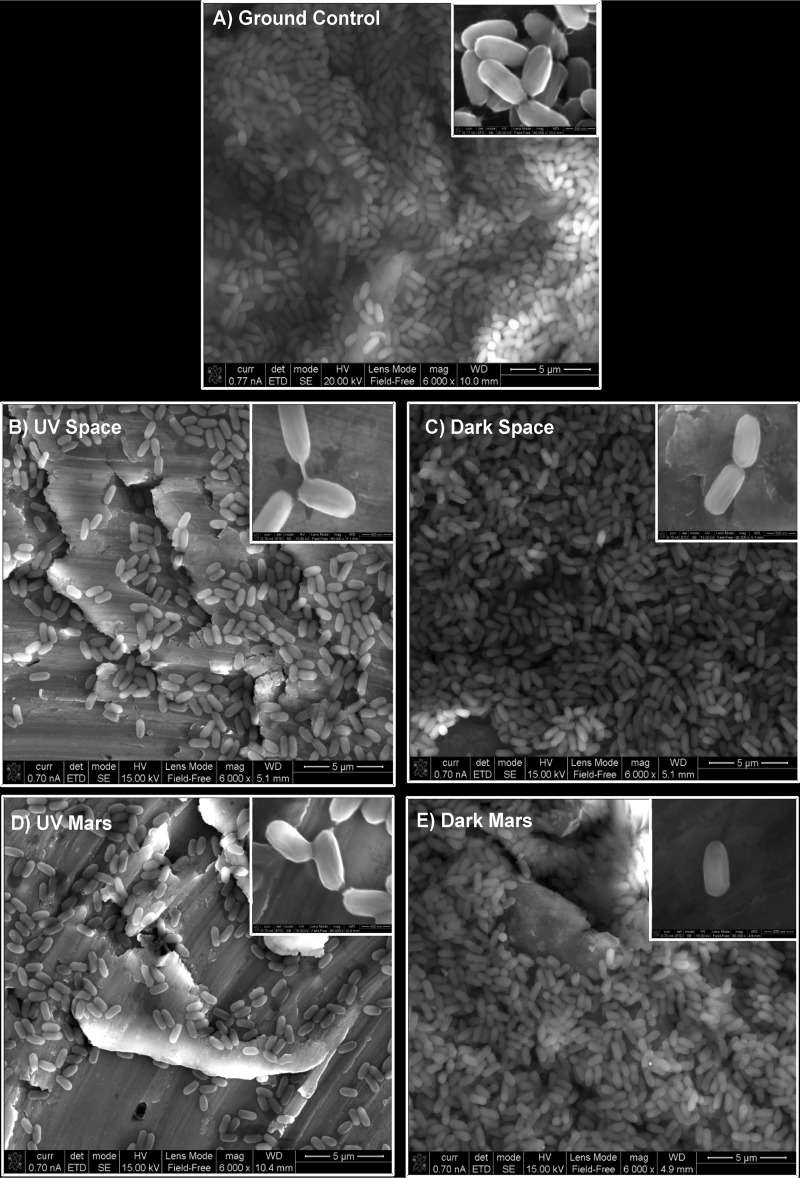
Field emission scanning electron micrographs of Bacillus pumilus SAFR-032 spores on aluminum coupons before and after an 18-month exposure to various space conditions. Intact spore structures are shown in the insets.

**FIG 2:**
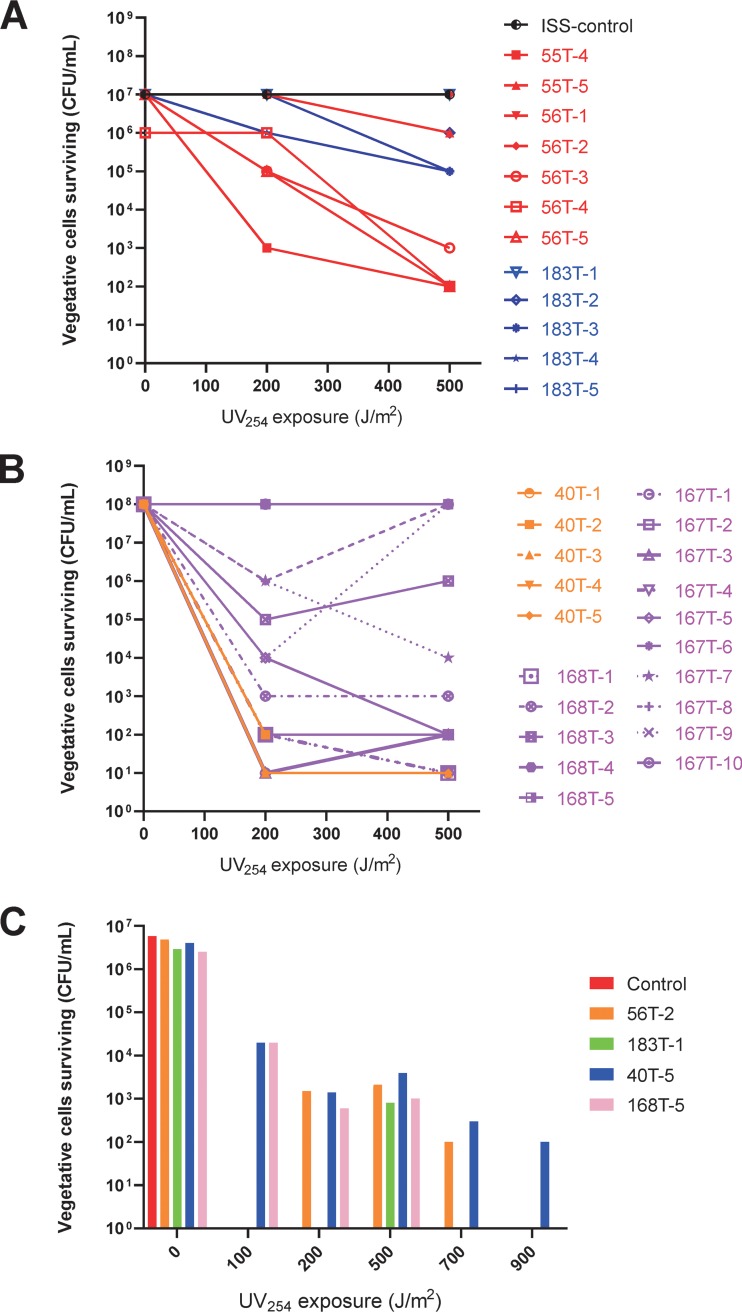
Measurement of UV_254_ (dosages of 0, 200, and 500 J/m^2^) resistance from a large collection of ISS survivors and in-depth measurement of UV_254_ resistance (dosages of 0 to 900 J/m^2^) of selected strains. (A) Vegetative cells of *B. pumilus* SAFR-032 that survived UV-Space were designated 55 series (2 strains) and 56 series (5 strains). Strains that survived UV-Mars were denoted as 183 series (5 strains). One parental ISS control strain kept on the ground and never flown was used for comparison. (B) Vegetative cells of *B. pumilus* SAFR-032 that survived Dark-Space were designated 40 series (5 strains). Strains that survived Dark-Mars were denoted as 167 series (10 strains) and 168 series (6 strains). (C) Quantitative measurement of UV_254_ resistance of selected *B. pumilus* cells. Cells with the following designations were used to measure UV resistance by exposure to UV_254_ at a dosage from 0 to 900 J/m^2^: Control (kept on the ground and never flown to space), UV-Space (56T-2), UV-Mars (183T-1), Dark-Space (40T-5), and Dark-Mars (168T-5). Measurements of two dilution series were plated and averaged.

The scanning electron microscope (SEM) micrographs of the spores exposed to various conditions are shown in Fig. 1. These micrographs were generated directly from the coupons after 18 months of exposure to space or kept on the ground. Even though the starting spore concentrations were equal in all the coupons made, the spore density was less under the UV-exposed conditions (Fig. 1B to D) compared to the ground control (Fig. 1A), as well as in Dark-exposed microorganisms (Fig. 1C to E). UV resistance was determined for 32 isolates that survived under various space conditions and were randomly selected, regrown, and subjected to various exposures to UV at 254 nm (UV_254_). The UV survival kinetics (0, 200, and 500 J/m^2^) of the vegetative cells of the UV-Space (7 strains), UV-Mars (5 strains), Dark-Space (5 strains), and Dark-Mars (15 strains) conditions are depicted in Fig. 2A (UV survivors) and Fig. 2B (Dark survivors). The results showed that the UV survival was strain specific and was not related to the exposure conditions (UV or Dark or Space or Mars). UV survivability of the selected UV-Space (56T-2), UV-Mars (183T-1), Dark-Space (40T-5), and Dark-Mars (168T-5) strains and the ground control was examined, and the strains were further subjected to UV_254_ exposure from 0 to 900 J/m^2^ (Fig. 2C). The vegetative cells of the ground control did not grow even at 25 J/m^2^, which was in concordance with UV growth kinetics of vegetative cells reported in other *Bacillus* species (8). Vegetative cells of first-generation cultures of space-surviving SAFR-032 strains exhibited only a 3-log reduction in viability at 500 J/m^2^ (Fig. 2C). In particular, the UV-Space 56T-2 strain grew until after 700 J/m^2^, and the Dark-Space 40T-5 strain was the lone UV survivor at a dose as high as 900 J/m^2^.

Since the genomes of all these SAFR-032 space-surviving strains did not show any variations or single nucleotide polymorphisms compared with the ground control, only the genome of SAFR-032 was deposited (NCBI accession no. CP000813.4) (2, 6). Lack of significant genomic changes emphasizes the importance of proteomic analyses to understand the molecular mechanisms of resistance to extreme irradiation conditions.

### Proteome analysis overview.

**FIG 3:**
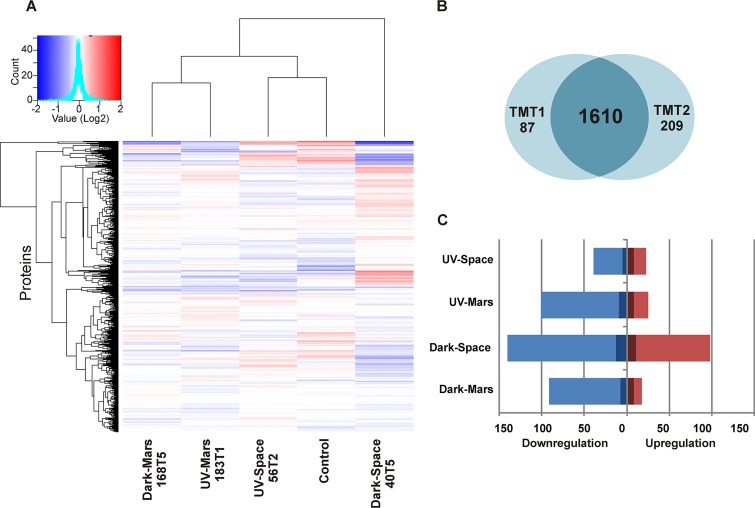
Summary of *B. pumilus* SAFR-032 proteins identified and quantified in all technical (*n* = 3) and biological (*n* = 2) replicates. (A) Heat map hierarchical clustering of proteins that have been quantified in each strain. The color key indicated the values that represented the protein abundance ratio (log_2_) and the number of proteins counted in each value. (B) Venn diagram representing the overlap of proteins between biological duplicates that were used for the statistical analysis: tandem mass tag (TMT) experiments 1 and 2. (C) Number of proteins up- or downregulated in abundance for each strain compared to the ground control (fold change of ≥±1.5). The dark blue/red colors represent proteins of differential abundance (*P* ≤ 0.05, one-way ANOVA).

The quantitative proteomic differences in the first-generation cultures of archived parent strains of the *B. pumilus* SAFR-032 ground control and the UV-Space (56T-2), UV-Mars (183T-1), Dark-Space (40T-5), and Dark-Mars (168T-5) strains were identified by using isobaric TMT labeling followed by liquid chromatography-multistage mass spectrometry (LC-MS/MS) analysis on an Orbitrap Fusion Tribid mass spectrometer. The LC-MS/MS spectra were searched against 3,601 proteins reported in NCBI database. In total, 2,146 and 2,225 proteins were identified in each biological replicate, accounting for approximately 60% of all proteins encoded in the *B. pumilus* SAFR-032 genome. These proteins were then filtered, and only proteins that were identified and quantified in all technical (*n* = 3) and biological (*n* = 2) replicates were selected for statistical analysis (1,610 proteins) (Fig. 3A and B; see Table S1 in the supplemental material).

10.1128/mSystems.00195-19.1 TABLE S1 Differential protein abundances in space-surviving strains in comparison with the ground control. Download Table S1, XLSX file, 0.07 MB. Copyright © 2019 Chiang et al. 2019 Chiang et al. This content is distributed under the terms of the Creative Commons Attribution 4.0 International license.

The protein abundance levels of four space-surviving strains were first normalized to the unexposed *B. pumilus* SAFR-032 strain (ground control), and the number of differentially abundant proteins of each strain are summarized in Fig. 3C. A total of 301 proteins were either increased or decreased in their abundance, at >1.5-fold change in at least one strain, and 70 of these proteins had a *P* value of ≤0.05 (one-way analysis of variance [ANOVA]). Heat map hierarchical clustering of proteins showed that the Dark-Space strain 40T-5 did not cluster with other strains, consistent with its unique protein abundance pattern (Fig. 3A). The Dark-Space strain contained the largest number of differentially abundant proteins (*n* = 238) compared to the UV-Space (*n* = 62), UV-Mars (*n* = 126), and Dark-Mars (*n* = 109) strains (Fig. 3C). Moreover, the Dark-Space strain had more unique proteins with differential abundances (*n* = 117) than the UV-Space (*n* = 17), UV-Mars (*n* = 21), and Dark-Mars (*n* = 9) strains (Table S1). The greatest change in protein abundance for the Dark-Space strain was consistent with its high levels of UV radiation resistance (Fig. 2C), suggesting that its particular proteome may be mechanistically involved in the adaptive stress response.

**FIG 4:**
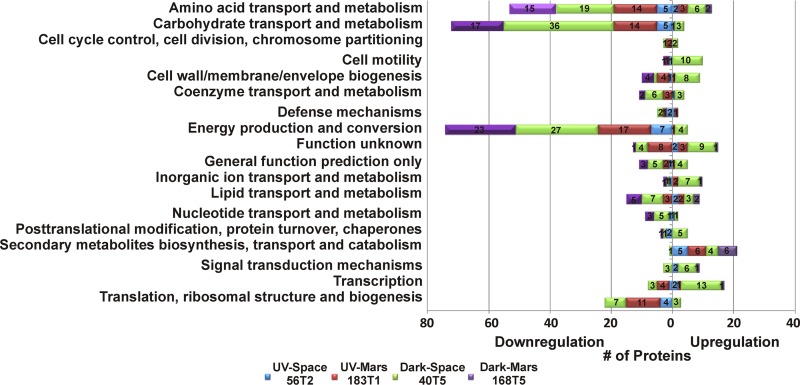
Dysregulated *B. pumilus* SAFR-032 proteins, classified according to COG categories (fold change of ≥±1.5).

Functional annotations of proteins were derived from the UniProt and the EggNOG version 4.5.1 (9, 10). Clusters of Orthologous Groups (COGs) were used to classify the differential abundance of proteins (Fig. 4; see Table S2 in the supplemental material). A large portion of downregulated proteins in all four space-surviving strains were involved in “carbohydrate transport and metabolism,” “energy production and conversion,” “amino acid transport and metabolism,” and “lipid transport and metabolism.” Nevertheless, several proteins that associated with “secondary metabolites biosynthesis, transport, and catabolism,” “transcription,” “cell motility,” and “signal transduction mechanisms” were upregulated and related to the cellular fitness and stress response.

10.1128/mSystems.00195-19.2 TABLE S2 COG categories of proteins with differential abundances. Download Table S2, XLSX file, 0.03 MB. Copyright © 2019 Chiang et al. 2019 Chiang et al. This content is distributed under the terms of the Creative Commons Attribution 4.0 International license.

### Alteration of proteins involved in catabolic pathways.

The differential proteome of four space-surviving strains showed a comprehensively downregulated abundance of proteins related to carbohydrate transport and energy production (Fig. 4; Table 1). Several proteins associated with phosphoenolpyruvate (PEP)-dependent sugar phosphotransferase system (PTS), including HPr phosphocarrier protein, glucose, fructose, sugar, β-glucoside, and lactose/cellobiose PTS transporter were downregulated compared to the ground control. Only the abundance of PTS sorbitol transporter IIA was increased in space-surviving strains. Enzymes related to the tricarboxylic acid (TCA) cycle, including α-ketoacid dehydrogenase, citrate synthase, 2-oxoglutarate dehydrogenase (2-ODH), dihydrolipoyllysine-residue succinyltransferase (DLST), dihydrolipoly dehydrogenase, etc., were also decreased in abundance in space-surviving strains compared to the control. Previously, gene expression levels of bacterial PTS, glucose, and non-glucose catabolism pathways were upregulated in response to limited nutrient supply and/or under space conditions (11–13). However, our data showed that the abundance of these proteins in all space-surviving strains that were recultured aerobically in rich Trypticase soy broth medium on Earth was either decreased or not significantly changed. The bacterial PTS is known to control carbohydrate uptake and is involved in bacterial carbon catabolite repression. By tightly controlling the expression and utilization of nonpreferred carbohydrates like sorbitol and maltose when growing in the presence of preferred carbons such as glucose and fructose, bacteria can optimize their metabolism for energy generation (14). Our data suggest that the space-surviving strains have a preference for using sorbitol as the carbon source, even though they were cultured in a glucose-rich medium. Alternatively, upregulation of the sorbitol transporter may be a stress response similar to what has been reported for other microorganisms (15).

**TABLE 1:** Dysregulated proteins involved in catabolic pathways

GI no., description^*a*^	Protein abundance ratio^*b*^				COG category^*c*^
	UV-Space	UV-Mars	Dark-Space	Dark-Mars	
Phosphoenolpyruvate-dependent sugar PTS					
500860925, PTS β-glucoside transporter subunit EIIBCA	−0.689	−1.311	−1.243	−0.787	CTM
500860804, PTS β-glucoside transporter subunit EIIBCA	−0.259	−0.378	−0.740	−0.235	CTM
500860752, PTS fructose transporter subunit IIA	−0.046	−0.509	−0.831	−0.705	CTM
500857289, PTS fructose transporter subunit IIB	−1.247	−1.819	−1.990	−1.138	CTM
500857288, PTS fructose transporter subunit IIC	−0.556	−0.483	−1.493	−0.717	CTM
500857290, PTS glucose transporter subunit IIA	−0.960	−1.063	−1.771	−0.907	CTM
500860749, PTS lactose/cellobiose transporter subunit IIA	−0.381	−0.294	−0.873	−0.442	CTM
500860751, PTS sugar transporter subunit IIB	−0.895	−0.963	−1.596	−0.471	CTM
489303627, multispecies, phosphocarrier protein HPr	−0.506	−1.019	−0.887	−0.451	CTM
494764325, multispecies, PTS sorbitol transporter subunit IIA	0.140	0.480	1.205	0.173	CTM
TCA cycle					
500857502, α-ketoacid dehydrogenase subunit β*	−0.625	−2.094	−3.439	−2.575	EPC
500858738, citrate synthase	0.227	−0.688	−1.332	−0.902	EPC
495621484, multispecies, citrate synthase	−0.125	−0.775	−1.589	−0.990	EPC
500858752, 2-oxoglutarate dehydrogenase subunit E1	−0.165	−0.346	−1.000	−0.666	EPC
500858751, dihydrolipoyllysine-residue succinyltransferase	−0.362	−0.676	−1.264	−0.810	EPC
489304636, multispecies, succinate-CoA ligase subunit α	−0.086	−0.563	−1.168	−0.541	EPC
500858441, multispecies, succinyl-CoA ligase subunit β	0.228	−0.354	−0.929	−0.597	EPC
500857504, dihydrolipoyl dehydrogenase	−0.770	−1.816	−2.652	−2.276	EPC
500859474, multispecies, phosphoenolpyruvate carboxykinase (ATP)	0.375	−0.832	−1.788	−0.960	EPC
Electron transfer chains					
489303411, cytochrome (ubi)quinol oxidase subunit III	−0.296	−0.570	−1.525	−1.170	EPC
754202327, cytochrome *c*′	0.348	−0.479	−0.641	−0.719	EPC
500858340, cytochrome *c* oxidase subunit I	0.028	−0.307	−1.355	−0.653	EPC
489304759, cytochrome *c* oxidase subunit II	−0.215	−1.312	−1.443	−1.435	EPC
494765503, multispecies, menaquinol-cytochrome *c* reductase iron-sulfur subunit	0.107	−0.922	−1.095	−0.759	EPC
500859318, electron transfer flavoprotein subunit alpha/FixB family protein	−0.518	−0.863	−1.327	−0.913	EPC
500859319, electron transfer flavoprotein subunit β*	−0.560	−0.979	−1.465	−0.925	EPC
500858688, (Fe-S)-binding protein*	−0.639	−0.931	−1.140	−1.195	EPC
754202216, NADH-dependent flavin oxidoreductase	−0.950	−0.626	−0.749	−0.673	EPC
Fatty acid beta-oxidation					
500859322, long-chain fatty acid-CoA ligase	−0.062	−0.677	−1.386	−0.756	LTM
500859693, 3-hydroxyacyl-CoA dehydrogenase	−0.281	−0.685	−1.361	−0.736	LTM

a *, *P* ≤ 0.05 (one-way ANOVA).

b The values shown represent protein abundance ratios (log_2_) normalized to the ground control.

c CTM, carbohydrate transport and metabolism; EPC, energy production and conversion; LTM, lipid transport and metabolism.

Metabolic shutdown of TCA cycle and electron transport chains has been demonstrated in both bacterial and human cell lines, which were exposed to oxidative stress (16, 17). In particular, decreasing the activity of 2-ODH controls the redox balance by diminishing reactive oxygen species (ROS) and NADH formation (16). The reduction of NADH also further reduces NADH-generated ROS via oxidative phosphorylation (18). In line with our observation, space-surviving strains showed decreased abundance of 2-ODH and its protein complex DLST compared to the ground control. Especially, the Dark-Space strain showed the lowest abundance of 2-ODH and DLST (2- and 2.4-fold lower than the ground control) compared to other strains. Furthermore, space-surviving strains also exhibited reduced abundance of proteins involved in electron transfer activity, including cytochrome *c* oxidase subunit, electron transfer flavoprotein subunit, NADH-dependent flavin oxidoreductase, etc. Particularly flavin is a redox agent and is involved in ROS formation (19). Decreased abundance of flavoproteins in space-surviving strains suggested the reduction of ROS generation. Also, the Dark-Space strain showed the lowest expression of electron transfer flavoprotein subunits alpha and beta (∼2.6-fold lower than the control) compared to other strains. In addition, beta-oxidation of fatty acid is known to generate acetyl coenzyme A (acetyl-CoA) as well as NADH and reduced flavin adenine dinucleotide (FADH_2_), which can further enter the TCA cycle and electron transport chain (20, 21). In line with the reduced protein abundance involved in the TCA cycle and electron transfer chain in space-surviving strains, several fatty acid beta-oxidation proteins such as long-chain fatty acid-CoA ligase and 3-hydroxyacyl-CoA dehydrogenase were also decreased in abundance, particularly in the Dark-Space strain (∼2.6-fold lower than the control). Collectively, the differential abundance of these proteins in space-surviving strains suggested that the regulation of redox signaling to prevent the generation of oxidants or ROS in cells might contribute to their survival in the extreme environment.

### Alteration of proteins contributed to the bacterial competitive growth advantage.

Living microorganisms are known to have complex but efficient mechanisms to adapt to a variety of environmental stresses for survival. The increased abundance of certain proteins might contribute to the growth advantage of the space-surviving strains. Those include nonribosomal peptide synthetase (NRPS), polyketide synthase (PKS), flagellin, and chemotaxis (Table 2). NRPS and PKS are multifunctional enzymes that synthesize a diverse group of secondary metabolites in bacteria and fungi. These secondary metabolites include toxins, antibiotics, siderophores, pigments, or immunosuppressants that could play an important role in the development, survival, and virulence of microorganisms (22). Even though the secondary metabolites that are produced by NRPS and PKS of *B. pumilus* are yet unknown, previous studies demonstrated that the production of secondary metabolites related to antimicrobial, antiviral activity, and biofilm formation were important for the survival and growth of B. subtilis and other bacilli (23, 24). In addition, both flagellin and chemotaxis proteins are involved in bacterial cell motility and are essential for bacteria to sense their surroundings and migrate toward more favorable environments. Several studies showed that flagellin and chemotaxis can be essential for bacterial adhesion, invasion, biofilm formation, and virulence (25). Upregulation of flagellin and chemotaxis genes was identified in bacteria that were cultured under limited nutrient and stress conditions (26–28). Notably, the Dark-Space strain expressed the highest abundance of NRPS, flagellin, and chemotaxis proteins (∼1.5- to 2-fold higher than the control), suggesting that it may have particular secondary metabolites profiles and cell motility ability to enhance its growth advantage.

**TABLE 2:** Dysregulated proteins involved in competitive growth advantage

GI no., description^*a*^	Protein abundance ratio^*b*^				COG category^*c*^
	UV-Space	UV-Mars	Dark-Space	Dark-Mars	
500857672, nonribosomal peptide synthetase*	0.730	0.840	1.157	0.920	SMB
500857673, polyketide synthase	0.790	0.813	0.627	0.812	SMB
500857674, polyketide synthase	0.879	0.739	0.557	0.793	SMB
500857675, polyketide synthase	0.958	0.764	0.472	0.825	SMB
500859561, chemotaxis protein	−0.278	0.110	0.655	−0.085	CM
500859564, chemotaxis protein	0.232	0.512	1.099	0.424	CM
500858269, chemotaxis protein CheV	−0.123	0.007	0.681	−0.116	CM
500858470, chemotaxis protein CheW	−0.051	0.150	0.776	0.076	CM
500858471, chemotaxis protein CheC	0.046	0.327	0.878	0.054	STM
500860007, methyl-accepting chemotaxis protein	0.128	0.392	0.730	0.221	CM
500859562, methyl-accepting chemotaxis protein	−0.062	0.111	1.024	0.202	CM
500858139, flagellin	0.140	0.263	0.654	0.499	CM
500858460, flagellar basal body-associated protein FliL	−0.452	−0.529	1.081	−0.437	CM
500858457, flagellar hook assembly protein FlgD	−0.684	−0.846	0.520	−0.669	CM
500858451, flagellar motor switch protein FliG	−0.093	0.160	0.614	−0.091	CM

a *, *P* ≤ 0.05 (one-way ANOVA).

b The values shown represent protein abundance ratios (log_2_) normalized to the ground control.

c SMB, secondary metabolite biosynthesis, transport and catabolism; CM, cell motility; STM, signal transduction mechanisms.

### Alteration of enzyme abundance involved in the stress response.

Several stress response-related proteins had increased abundance in space-surviving strains compared to the ground control, including sporulation protein (Spo0M), universal stress protein (UspA), and stress response transcriptional regulators (Table 3). One major factor that contributes to the resistance and long-term survival of *Bacillus* may likely be its spore-forming capability. Enhanced sporulation of space-surviving strains was observed compared to the ground control (6). Here, our data showed that Spo0M, an important sporulation regulator that is involved in initiating sporulation and cell division (29), and dihydropteridine reductase or cell division protein DivIB, an essential protein for efficient sporulation under harsh conditions (30), were increased in abundance in space-surviving strains, particularly in the Dark-Space strain (∼1.7- to 2-fold higher than the control). Moreover, the primary function of UspA is to protect bacteria from environmental stresses such as starvation, oxidative stress, chemical stress, etc. (31). UspA cooperates with RecA to protect against DNA damage, and deletion of UspA can result in sensitivity to UV exposure (32). The increased abundance of UspA in space-surviving strains, particularly in the UV-Space (2.5-fold) and Dark-Space (1.6-fold) strains, suggested that UspA might contribute to the enhanced resistance in these strains. In addition, the abundance of other stress response regulatory proteins such as cold shock protein (Csp) and ECF RNA polymerase sigma factor SigM were also increased in space-surviving strains compared to the ground control. The induction of bacterial Csp was shown as a response to the downshift of temperature as well as to other stressors, such as starvation and osmotic stress (33, 34). Furthermore, the expression of SigM is induced in response to acid, heat, salt, superoxide, and cell envelope stresses, and SigM is essential in maintaining bacterial membrane and cell wall integrity (35, 36).

**TABLE 3:** Dysregulated proteins involved in the stress response

GI no., description^*a*^	Protein abundance ratio^*b*^				COG category^*c*^
	UV-Space	UV-Mars	Dark-Space	Dark-Mars	
500859623, sporulation protein	0.160	0.323	0.775	0.248	CCC
500858367, dihydropteridine reductase	−0.550	0.213	0.999	−0.097	CCC
754201850, universal stress protein	1.328	0.662	0.664	0.437	STM
500858196, organic hydroperoxide resistance protein	−0.146	−0.087	−1.051	−0.329	DM
500857905, catalase	−0.393	−0.535	−1.297	−0.844	IIT
495622186, multispecies, cold shock protein	0.562	−0.208	0.872	0.071	Transcription
500859389, MarR family transcriptional regulator*	1.121	0.122	0.310	−0.082	Transcription
500857308, multispecies, MarR family transcriptional regulator	0.098	0.249	0.884	−0.004	Transcription
489304493, multispecies, ECF RNA polymerase sigma factor SigM*	0.055	0.348	0.518	0.535	Transcription

a *, *P* ≤ 0.05 (one-way ANOVA).

b Values represent protein abundance ratios (log_2_) normalized to the ground control.

c CCC, cell cycle control, cell division, chromosome partitioning, transport, and catabolism; UN, function unknown; STM, signal transduction mechanisms; DM, defense mechanism; IIT, inorganic ion transport and metabolism.

Interestingly, some known stress response proteins such as organic hydroperoxide resistance protein (Ohr), and catalase were downregulated (Table 3), while others, such as superoxide dismutase (SOD), were not significantly changed in the space-surviving strains (see Table S3 in the supplemental material) (37, 38). Decreased abundance of these proteins might be mediated by the stress response regulator MarR. Mar is the multiple antibiotic resistance protein regulator that regulates the expression of genes involved in stress responses, virulence, or degradation or resistance to toxic chemicals (39). Mar repressor (MarR) functions as a negative regulator of the Mar operon. The expression of MarR inhibits the effect of bacterial resistance to antibiotics and synthesis of antioxidants, but this repressive effect can be reversed when bacteria sense cellular stress from the environment (40–42). Since MarR is known to regulate several stress response proteins such as Ohr regulator and SOD (43, 44), increased abundance of MarR, especially in the Dark-Space (∼1.5-fold) and UV-Space (∼1.6-fold) strains, might functionally explain the decreased abundance of stress response proteins observed in our study. Since the proteome generated in this study was from the first-generation cells grown on Earth without additional stress exposure, it would be interesting to investigate proteomic changes before and after stresses (e.g., UV exposure), to identify the proteins that are directly in response to the stress conditions. Collectively, increased abundance of stress response proteins in Dark-Space and UV-Space strains might contribute to their resistance to oxidative stress associated with the extreme space environments.

10.1128/mSystems.00195-19.3 TABLE S3 List of all proteins identified and quantified. Download Table S3, XLSX file, 0.2 MB. Copyright © 2019 Chiang et al. 2019 Chiang et al. This content is distributed under the terms of the Creative Commons Attribution 4.0 International license.

### Validation of differences in protein abundance using enzyme activity and ATP assays.

**FIG 5:**
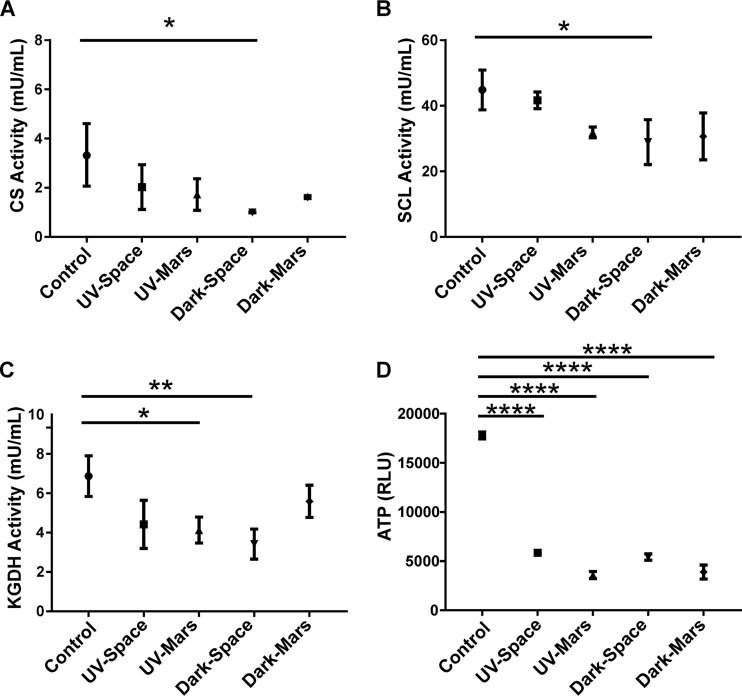
Validation of proteomics data through enzymatic assays and ATP determination. (A to C) Citrate synthase (CS), succinyl-CoA ligase (SCL), and α-ketoglutarate dehydrogenase (KGDH) enzymatic activities were measured by using 25, 5, and 100 μg total protein from each strain. (D) Amount of ATP in lysate with 10 μg total protein per strain. Asterisks represent a *P* value of ≤0.05 (*), ≤0.005 (**), or ≤0.0001 (****) compared to the ground control as determined by one-way ANOVA with Tukey’s test for multiple comparisons.

Consistent with previous observations in the stress response of bacteria (16, 17, 45), our proteomics analysis showed downregulation of several proteins involved in the TCA cycle and electron transfer chain in space-surviving strains compared to the ground control. To functionally validate the results, we measured the enzymatic activities of key enzymes in the TCA cycle, including citrate synthase (CS), succincyl-CoA ligase (SCL), α-ketoglutarate dehydrogenase (KGDH or 2-ODH), and the production of ATP. All three enzymatic assays showed increased activity in the ground control strain compared to the space-surviving strains (Fig. 5A to C). In particular the Dark-Space strain showed a significantly lower enzymatic activity compared to that of the ground control which was consistent with its protein profiles. Moreover, the amount of ATP production was significantly reduced in all space-surviving strains compared to that of the ground control (Fig. 5D). The decreased production of ATP in all space-surviving strains was also in agreement with our proteomics analysis in which proteins associated with energy production and conversion pathways had reduced abundance. Taken together, these functional assays demonstrated that the activities of TCA cycles and energy metabolism were decreased in space-surviving strains compared to the ground control, which was consistent with the observed quantitative changes in the proteomes.

### Conclusions.

This study provides the first comprehensive picture of the proteomic changes in *B. pumilus* SAFR-032 induced by exposure to outer space. It was expected that the abundances of DNA repair proteins and some other stress response proteins were not significantly changed in bacterial cultures without stress exposure. Nevertheless, these data suggest that the abundance of metabolic enzymes in vegetative *B. pumilus* SAFR-032 strains has been altered after 18 months of space exposure of its spores even after regrowing them on Earth. The altered protein abundances in the categories of energy metabolism and stress response suggest that the space-surviving strains, in particular the Dark-Space strain, may have evolved to better cope with stressful environments. Future epigenetic studies should be undertaken to clarify the underlying mechanism that caused the observed proteomic alterations. Further multiomics experiments should be conducted to examine various stressors (e.g., irradiation, oxidative stress conditions, etc.) at different time points to understand the molecular pathways for their resistance in extreme environments.

## MATERIALS AND METHODS

### Bacterial culture and selection of UV_254_ resistance.

Among the surviving spores under UV-Space (7 strains), UV-Mars (5 strains), Dark-Space (5 strains), and Dark-Mars (15 strains) conditions, 32 strains were regrown after returning to Earth and tested for their UV survival. One strain from each of these four conditions was selected for its highest UV survival for subsequent experimentations. Cells of *B. pumilus* SAFR-032 (control and space-surviving strains) were grown in Trypticase soy broth overnight and diluted in phosphate-buffered saline (pH 7.2) to achieve a density of 0.1 at the optical density at 600 nm (OD_600_). Initial cell density (10^6^ to 10^9^ CFU/ml) was estimated by serial dilution plating before each exposure. A low-pressure handheld mercury arc UV lamp (UVP, Inc.; model UVG-11; [UVC, 254 nm]) was placed at a fixed height over the sample, and the UV flux at the surface of the spore suspension was measured with a UVX digital radiometer (UVP, Inc.). Exposure times necessary to yield fluences from 200 to 4,000 J/m^2^ at the sample surface were determined (UV flux was 1 J/m^2^ s^2^). Under aseptic conditions, each cell suspension was placed into an uncovered 50-mm glass petri dish, within a class II biological safety cabinet fitted with UV lighting (Fisher Scientific), and stirred with a magnetic stir bar (3 to 5 mm in length) while being exposed to UV irradiation. When 10 ml (10^6^ CFU/ml) of cell suspension was added to the 50-mm petri dish, the height of the liquid was 3 mm. The liquid spore suspension was stirred gently (100 rpm) to avoid splashing; this setup allowed the cells to be exposed to UVC evenly. From these, sample volumes of 100 μl were removed at specific time points, serially diluted, and spread atop tryptic soy agar (Difco) plates. After 24 to 48 h of incubation, the resulting CFU were counted, and the surviving fraction was calculated.

### Electron microscopy.

Field emission scanning electron microscopy (FE-SEM) provides high spatial resolution combined with low electron beam accelerating voltage. The low-beam voltage of the FE scanning electron microscope allows examination of electrical insulators without having to deposit a surface-conducting (carbon or metal) layer to eliminate specimen charging, which can lead to a distorted and often completely unusable image. The deposition of a conducting material to control charging can complicate the analysis. In many situations, a low electron beam voltage intrinsically results in a much sharper image, especially of thin structures composed of elements of low atomic number. A Philips (FEI, Hillsboro, OR) FE-SEM (XL-50) was used to analyze a majority of the samples. Elemental analysis is possible in a scanning electron microscope equipped with an energy-dispersive X-ray (EDX) analyzer. EDX is based on the analysis of the characteristic X rays emitted when an electron beam is incident on a sample. The acceleration voltage for analyzing aluminum samples was ∼10 to 20 kV. In the high-vacuum mode, secondary electron images were acquired. Similar settings were maintained when different models or scanning electron microscope instruments were used.

### Protein extraction.

Selected *B. pumilus* SAFR-032 strains that exhibited high UV_254_ resistance and were exposed to UV-Space (56T-2), UV-Mars (183T-1), Dark-Space (40T-5), or Dark-Mars (168T-5) conditions and the ground control were cultured under aerobic conditions in 300 ml of sterile Trypticase soy broth medium at 100 rpm at 25°C. After 48 h, the bacterial cultures were centrifuged at 4°C. The cell pellets were washed, and decanted pellets were stored at −80°C until further processing.

About 1 g of cell pellets was lysed with a buffer consisting of 100 mM triethylammonium bicarbonate (TEAB) with 1:100 Halt protease inhibitor cocktail (Thermo Fisher Scientific, Rockford, IL) and 1 mM phenylmethylsulfonyl fluoride (Sigma-Aldrich, St. Louis, MO). The crude homogenates were subjected to a Precellys 24 homogenizer (Bertin, Rockville, MD) in which each sample was processed inside a 2-ml cryotube with 1.0-mm glass beads three times at 4°C, at 6,500 rpm, for 1 min and 3 times with 15-s pauses in between). The lysed bacterial cells were centrifuged at 17,000 × g for 15 min. Protein concentrations in the supernatants were measured by Bradford assay (Bio-Rad Laboratories, Inc., Hercules, CA).

### TMT labeling.

Samples were processed as described by Romsdahl et al. with modification (46). Two hundred micrograms of proteins from each sample was precipitated in 20% trichloroacetic acid at 4°C. Protein pellets were obtained by centrifugation (17,000 × *g*), washed with ice-cold acetone, and resuspended in 25 μl TEAB (100 mM) and 25 μl 2,2,2-trifluoroethanol (TFE). Proteins were reduced by adding 1 μl of tris(2-carboxyethyl)phosphine (TCEP; 500 mM) and incubated for 1 h at 37°C (10 mM final TCEP concentration). Proteins were alkylated in the presence of iodoacetamide (IAA; 30 mM) in the dark for 1 h at room temperature. Mass spectrometry-grade trypsin/lysC (Promega, Madison, WI) at 2.5 μg per sample was used to digest the peptides overnight at 37°C.

The digested peptides were quantified using the Pierce quantitative colorimetric peptide assay (Thermo Fisher Scientific). Forty micrograms of peptides from each sample was labeled with the Thermo Scientific TMTsixplex (TMT^6^) isobaric mass tagging kit (ground control with TMT^6^-126, UV-Space with TMT^6^-127, UV-Mars with TMT^6^-128, Dark-Space with TMT^6^-129, and Dark-Mars with TMT^6^-130) according to the manufacturer’s protocol. The TMT^6^-131 label was used as a reference that contained 8 μg of peptides from each of the five samples. All six labeled-peptide mixtures were combined into a single tube, mixed, and fractionated using the Pierce high-pH reversed-phase peptide fractionation kit (Thermo Fisher Scientific). Eight fractions were dried using a SpeedVac concentrator and resuspended in 1% formic acid prior to LC-MS/MS analysis.

### LC-MS/MS analysis.

The samples were analyzed on an Orbitrap Fusion Tribrid mass spectrometer with an EASY-nLC 1000 liquid chromatograph, a 75-μm by 2-cm Acclaim PepMap100 C_18_ trapping column, and a 75-μm by 25-cm PepMap rapid-separation liquid chromatography (RSLC) C_18_ analytical column, and an Easy-Spray ion source (Thermo Fisher Scientific). The column temperature was maintained at 45°C, and the peptides were eluted at a flow rate of 300 nl/min over a 110-min gradient, from 3 to 30% solvent B (100 min), 30 to 50% solvent B (3 min), 50 to 90% solvent B (2 min), and 90% solvent B (2 min). Solvent A was 0.1% formic acid in water, and solvent B was 0.1% formic acid in acetonitrile.

The full MS survey scan (*m*/*z* 400 to 1,500) was acquired in the Orbitrap at a resolution of 120,000 and with an automatic gain control (AGC) target of 2 × 10^5^. The maximum injection time for MS scans was 50 ms. Monoisotopic precursor ions were selected with charge states 2 to 7 with a ±10-ppm mass window using a 70-s dynamic exclusion. The MS^2^ scan (*m*/*z* 400 to 2,000) was performed using the linear ion trap with the collision-induced dissociation (CID) collision energy set to 35%. The ion trap scan rate was set to “rapid,” with an AGC target of 4 × 10^3^ and a maximum injection time of 150 ms. Ten fragment ions from each MS^2^ experiment were subsequently simultaneously selected for an MS^3^ experiment. The MS^3^ scan (*m*/*z* 100 to 500) was performed to generate the TMT reporter ions in the linear ion trap using HCD at a collision energy setting of 55%, a rapid scan rate, an AGC target of 5 × 10^3^, and a maximum injection time of 250 ms.

### Quantitative proteomics analysis.

All MS/MS spectra were analyzed using the Proteome Discoverer (version 2.2.0.388; Thermo Fisher Scientific) with the Sequest-HT searching engines and a Bacillus pumilus SAFR-032 database containing 3,601 sequences (NCBI). The search was performed with the following parameters: a maximum of 2 missed cleavage sites, a minimum peptide length of 6, a tolerance for precursor ion masses of 5 ppm, and a tolerance for fragment ion masses of 0.6 Da. The static modification settings included carbamidomethyl of cysteine residues, and dynamic modifications included oxidation of methionine, TMT6plex modification of lysine ε-amino groups and peptide N termini, and acetyl modification of protein N terminus. A false-discovery rate (FDR) of 1% for peptides and proteins was obtained using a target-decoy database search. The reporter ion’s integration tolerance was 0.5 Da, while the coisolation threshold was 75%. The average signal-to-noise threshold of all reporter peaks was greater than 10. The quantitative abundance of each protein is determined from the total intensity of the detected reporter ions. The ratios between reporter and the reference reporter ion (TMT^6^-131) were used to estimate the abundance ratio of each protein.

Technical triplicate measurements for each protein were averaged. Only proteins that were identified and quantified with at least one peptide detected in all three technical replicates were considered for the analysis. The normalization across two biological sample sets in two separate TMT experiments was carried out according to Plubell et al., with modifications (47). Briefly, the data from the two TMT experiments were first corrected for small systematic differences resulting from sample loading variations and labeling efficiency, by normalizing the reporter ion totals for each channel. The trimmed mean of M value (TMM) normalization corrected the compositional bias by aligning the median of the distribution of abundance intensities between samples (48). Internal reference scaling was used to adjust the two TMT data sets onto the same intensity scale. The normalized data were then averaged and log_2_ transformed. One-way ANOVA was preformed to identify proteins that were significantly differentially expressed among strains (*P* ≤ 0.05). The identified proteins were also evaluated for up- and downregulation by setting a minimum limit of ±1.5-fold change.

### Validation of proteome analysis.

Citrate synthase, succinyl-CoA synthetase, and α-ketoglutarate dehydrogenase enzyme activities were measured using the assay kits according to the manufacturer’s protocols (Sigma-Aldrich; MAK193, MAK217, and MAK189). Briefly, each parental strain was cultured in Trypticase soy broth medium for 48 h. The bacterial cell pellets were collected and lysed using the specific assay buffer provided in each kit. The amount of total protein in each strain was measured using the bicinchoninic acid (BCA) protein assay (Pierce/Thermo Scientific) and used as a reference to compare enzyme activities between samples. One-way ANOVA with Tukey’s test for multiple comparisons was used to examine differences in enzyme activities between ground control and space-surviving strains.

The ATP production in each strain was measured by using CellTiter-Glo luminescent cell viability assay according to the manufacturer’s protocol (Promega; G7571). Briefly, each strain was cultured and lysed as described previously above. Lysates with 10 μg total protein per sample were used to compare each sample-specific amount of ATP. One-way ANOVA with Tukey’s test for multiple comparisons was used to analyze the statistical significance of ATP production between ground controls and space-surviving strains.

### Accession number(s).

The mass spectrometry proteomics data have been deposited into the ProteomeXchange Consortium via the PRIDE (49) partner repository under data set identifier PXD011292.
